# Histone H2A.X phosphorylation and Caspase-Initiated Chromatin Condensation in late-stage erythropoiesis

**DOI:** 10.1186/s13072-021-00408-5

**Published:** 2021-07-30

**Authors:** Nazish N. Jeffery, Christina Davidson, Scott A. Peslak, Paul D. Kingsley, Yukio Nakamura, James Palis, Michael Bulger

**Affiliations:** 1 Center for Pediatric Biomedical Research, Department of Pediatrics, University of Rochester, Rochester, NY USA; 2 Wilmot Cancer Institute, Department of Biomedical Genetics, University of Rochester, Rochester, NY USA; 3 Department of Medicine, Division of Hematology/Oncology, Hospital of the University of Pennsylvania, Philadelphia, PA USA; 4 Division of Hematology, The Children’s Hospital of Philadelphia, Philadelphia, PA USA; 5 Cell Engineering Division, RIKEN BioResource Center, Tsukuba, Ibaraki Japan

**Keywords:** H2A.X, g-H2A.X, BAZ1B, WSTF, Chromatin condensation, Erythropoiesis, Terminal erythroid maturation, Caspase, Phosphorylation, Apoptosis

## Abstract

**Background:**

Condensation of chromatin prior to enucleation is an essential component of terminal erythroid maturation, and defects in this process are associated with inefficient erythropoiesis and anemia. However, the mechanisms involved in this phenomenon are not well understood. Here, we describe a potential role for the histone variant H2A.X in erythropoiesis.

**Results:**

We find in multiple model systems that this histone is essential for normal maturation, and that the loss of H2A.X in erythroid cells results in dysregulation in expression of erythroid-specific genes as well as a nuclear condensation defect. In addition, we demonstrate that erythroid maturation is characterized by phosphorylation at both S139 and Y142 on the C-terminal tail of H2A.X during late-stage erythropoiesis. Knockout of the kinase BAZ1B/WSTF results in loss of Y142 phosphorylation and a defect in nuclear condensation, but does not replicate extensive transcriptional changes to erythroid-specific genes observed in the absence of H2A.X.

**Conclusions:**

We relate these findings to Caspase-Initiated Chromatin Condensation (CICC) in terminal erythroid maturation, where aspects of the apoptotic pathway are invoked while apoptosis is specifically suppressed.

**Supplementary Information:**

The online version contains supplementary material available at 10.1186/s13072-021-00408-5.

## Introduction

Erythropoiesis is a process of enormous magnitude, with the adult producing over 2 million red blood cells each second [1]. The generation of a functional erythrocyte from a committed progenitor cell requires significant changes in gene expression during a time of hemoglobin accumulation, rapid cell division and nuclear condensation. In the span of 3–4 cell divisions, erythroid cells condense their nuclei to approximately one-tenth of their original volume prior to enucleation [2]. Disruption of nuclear condensation is associated with myelodysplastic syndromes and congenital anemias [3, 4]. The factors and pathways that regulate the compaction of the erythroid genome, however, are poorly understood.

In addition to nuclear condensation and enucleation, terminal erythroid maturation is characterized by loss of organelles. These features are similar to events that occur during apoptosis subsequent to caspase activation. Caspase-3 in particular has been implicated in terminal erythroid maturation, and has been shown to cleave Acinus, which functions in apoptotic chromatin condensation, and lamin B [5–9]. However, radiation studies performed in the murine hematopoietic system have revealed that erythroid maturation is associated with a dramatic change in the ability of cells to apoptose. Late erythroid maturation is conventionally divided into distinct stages that are separated by cycles of cell division and distinguished by morphology [10, 11]. Thus, earlier erythroid progenitors (BFU-E and CFU-E) and the most immature erythroid precursor, proerythroblast (ProE), are readily induced to apoptose following clastogenic injury. However, the subsequent stages of maturation, basophilic, polychromatic and orthochromatic erythroblasts (BasoE, PolyE, OrthoE), are resistant to apoptotic cell death [10, 11]. This is correlated with high expression of apoptosis inhibitor Bcl-xL at these later stages [12, 13]. Thus, while erythroid maturation invokes components of a caspase-mediated apoptotic pathway, apoptosis is specifically inhibited.

Normal erythroid maturation requires the histone H2A variant, H2A.X [14]. Loss of histone H2A.X in erythroid cells in mice leads to a decrease in enucleated cells and dyserythropoiesis, along with the appearance of Howell–Jolly bodies, which are nuclear remnants indicative of inefficient enucleation, in mature red blood cells. Collectively, these features resemble phenotypes in patients with myelodysplastic syndrome and thus indicate defects in normal erythroid maturation [14]. While the best studied function of histone H2A.X is its role in amplifying the signal for DNA repair upon phosphorylation of S139 in its C-terminal tail region (H2A.X pS139 or γ-H2A.X), other functions have been described [15–19]. H2A.X has been implicated in the maintenance of genomic integrity, in condensation of chromatin during spermatogenesis and in silencing of embryonic genes in ES cells [15–17, 20, 21]. Histone H2A.X phosphorylation has also been implicated in apoptotic pathways. DNA damage is thought to induce dephosphorylation of H2A.X C-terminal tyrosine 142 (Y142) subsequent to phosphorylation of S139 (γ-H2A.X). In the absence of Y142 dephosphorylation, or if Y142 is re-phosphorylated, the dual phosphorylation can be recognized by apoptotic signaling factors [22, 23].

In this study, we find that loss of histone H2A.X in erythroid cells results in improper terminal maturation characterized by defective nuclear maturation and dysregulation of erythroid gene expression. In addition, murine erythroid cells exhibit increased γ-H2A.X foci at the basophilic erythroblast stage while also exhibiting Y142 phosphorylation. We generated knockout (KO) cell lines and performed transcriptome profiling in order to study the effects the loss of H2A.X or the Y142 kinase, BAZ1B, have on erythroid maturation. These data suggest that histone H2A.X serves multiple roles in late-stage erythropoiesis, with phosphorylation of S139 and Y142 occurring upstream of caspase-initiated chromatin condensation (CICC).

## Results

### Loss of H2A.X results in changes in chromatin condensation during maturation

To better understand the role of the variant histone H2A.X in erythropoiesis, we examined the erythroid compartment in bone marrow of H2A.X KO mice [24]. These mice exhibit macrocytic anemia with decreased numbers of reticulocytes, indicative of reduced production of RBCs; these phenotypic changes are accompanied by increased cell size (mean corpuscular volume, MCV) compared to WT mice (Additional file 2: Fig. S1a, b). Cell numbers for other hematopoietic lineages are not significantly affected (Additional file 2: Fig. S1a), indicating that H2A.X loss specifically impacts the erythroid lineage. Next, we quantified erythroblast intermediates in bone marrow (Additional file 2: Fig. S1c) [25] and found an increase in total Ter119+ cells compared to WT (Additional file 1: Fig. S1d), consistent with an increased erythropoietic drive in response to anemia. In addition, H2A.X KO mice have an increased proportion of more immature (basophilic) erythroblasts in the bone marrow and a corresponding decrease in the proportion of the most mature (orthochromatic) erythroblasts (Additional file 1: Fig. S1e), indicating potential defects in terminal erythroid maturation. Taken together, these data demonstrate that loss of H2A.X leads to dysregulated terminal maturation and an inability to maintain steady-state levels of erythrocyte production.

Terminal maturation of erythroid precursors is characterized by a substantial decrease in nuclear size prior to enucleation, associated with chromatin condensation. Increased MCV results in defects in nuclear maturation, as seen in megaloblastic anemias due to folate or B12 deficiencies [26]. Thus, the increased MCV of erythroblasts in H2A.X KO mice may reflect improper nuclear maturation. Consistent with this, we find that basophilic erythroblasts exhibit a higher nuclear:cytoplasmic (N:C) ratio compared to WT (Additional file 2: Fig. S1f), suggesting that nuclear maturation in H2A.X KO erythroid cells is impaired. Taken together, these data indicate that histone H2A.X plays a role in normal erythropoiesis, and may be required for normal nuclear maturation and chromatin condensation.

### Prevalence of H2A.X and phosphorylated H2A.X in terminal erythroid maturation

**Fig. 1:**
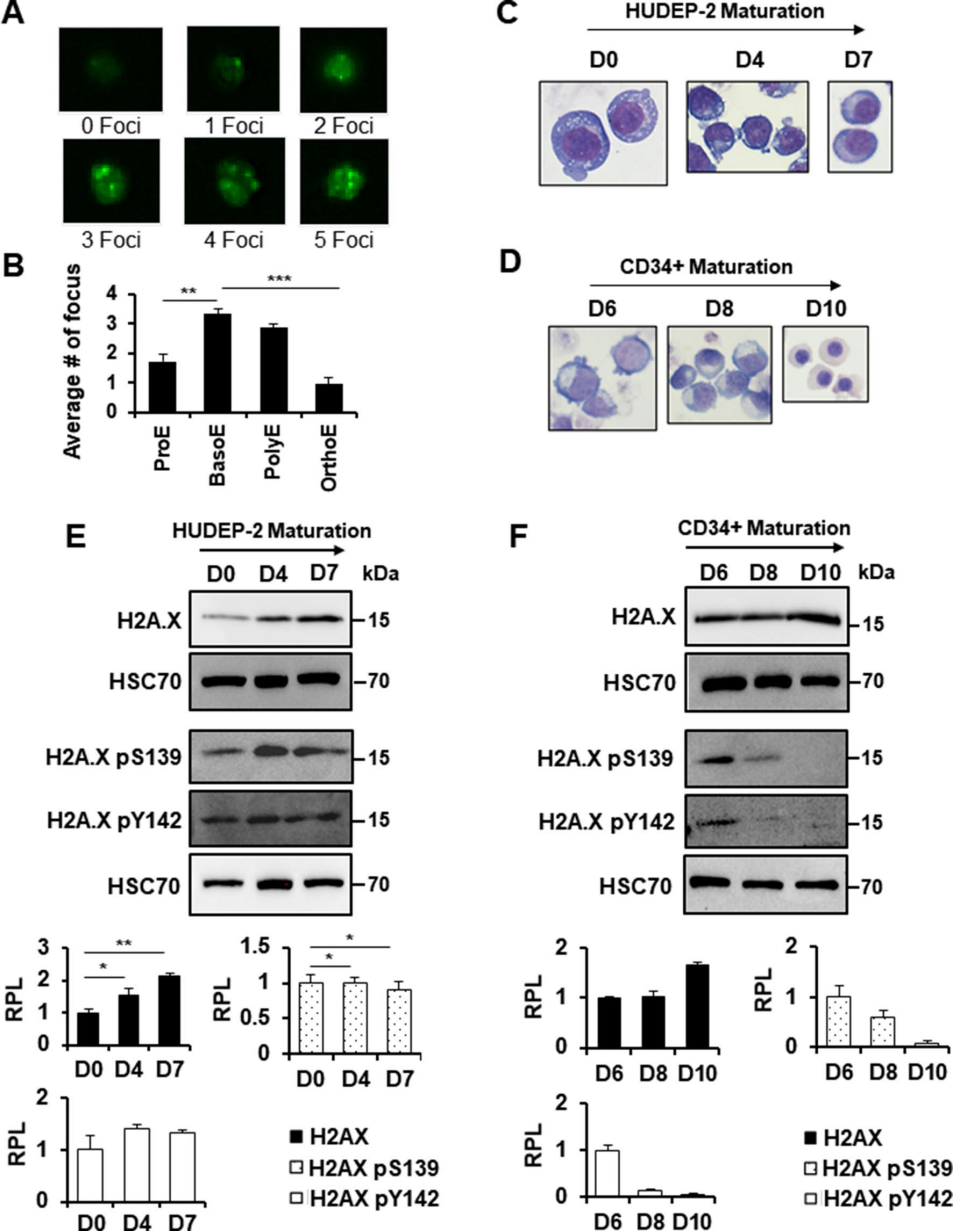
Presence of histone H2A.X and γ-H2A.X during terminal erythroid maturation. Imaging (**A**) and quantification (**B**) of γ-H2A.X foci in sorted murine bone marrow (*n* = 4). Giemsa staining of WT (**C**) HUDEP-2 cells at D0, D4, and D7 of maturation and **D** WT human CD34+ progenitor cells at D6, D8, and D10 of maturation after undergoing CD36 synchronization. Western blot analysis of histone H2A.X, γ-H2A.X (H2A.X pS139), and H2A.X pY142 during maturation in **E** HUDEP-2 (*n* = 3) and **F** human CD34+ progenitor (*n* = 2). HSC70 was used as a loading control. Quantification was performed using ImageJ. Signal obtained at the earliest time point measured was set at 1.0. Error bars represent ± SEM. Asterisks (*) indicate significance as follows: *p ≤ 0.05; **p ≤ 0.01. *p*-values were calculated with a two-tailed Student’s *t*-test

Phosphorylation of histone H2A.X at S139 (γ-H2A.X) is an early signal for DNA repair pathways in response to DNA breaks. To determine if γ-H2A.X is present during erythropoiesis, we performed antibody staining on cells isolated from adult murine bone marrow and subjected the cells to imaging flow cytometry as previously described (Additional file: Fig. S2) [25, 27]. We observed γ-H2A.X during terminal erythroid maturation, with the most abundant signal in basophilic erythroblasts (Fig. 1A, B). Intriguingly, this stage of erythropoiesis corresponds with increased resistance to cell death and the start of chromatin condensation.

To further investigate H2A.X in erythropoiesis, we examined histone H2A.X protein expression in two human in vitro models of erythroid differentiation: primary human CD34+ progenitor cells and the HUDEP-2 erythroid cell line. Proliferating HUDEP-2 cells appear to model basophilic erythroblasts, both by morphology and by gene expression; under maturation culture conditions, the cells gradually exhibit morphologies characteristic of later stages of erythropoiesis. For the purposes of this study, we examined HUDEP-2 cells at day 4 and day 7 of maturation, corresponding to a transition of the majority of the culture from BasoE to PolyE (Fig. 1C) [28]. For the primary cell cultures, we utilized an established protocol involving synchronization by selection of CD36+ erythroid progenitors [29]. In these cultures, morphology observed at days 6, 8, and 10 after synchronization corresponds with BasoE, PolyE and OrthoE, respectively (Fig. 1D) [29].

Through immunoblotting, we found that H2A.X protein levels increase during terminal erythroid maturation in both maturation models (Fig. 1E, F). Furthermore, the immunoblots indicate that γ-H2A.X dynamics are substantially different. However, while HUDEP-2 cells exhibit consistent levels of γ-H2A.X across the time course, primary cells show initially high levels that decrease dramatically after the basophilic erythroblast stage. Together these data indicate that H2A.X and γ-H2A.X exhibit distinct dynamics during erythropoiesis and may play a role in nuclear maturation.

In addition to its well-documented role in DNA repair, γ-H2A.X has been implicated as a signal for apoptosis when accompanied by phosphorylation of the nearby C-terminal Y142 (H2A.X pY142) [22, 23]. Given the occurrence of γ-H2A.X during erythroid maturation, we determined if H2A.X pY142 was also present by immunoblotting (Fig. 1E, F). H2A.X pY142 was present in both HUDEP-2 and primary cell cultures at varying levels throughout maturation (Fig. 1E, F). Interestingly, H2A.X pY142 was only evident at D6 of maturation in the primary cultures, corresponding to the basophilic erythroblast stage of maturation (Fig. 1F). These data suggest that H2A.X may be dynamically phosphorylated at both S139 and Y142 during erythroid maturation.

### Loss of H2A.X perturbs the expression of erythroid-specific genes

**Fig. 2:**
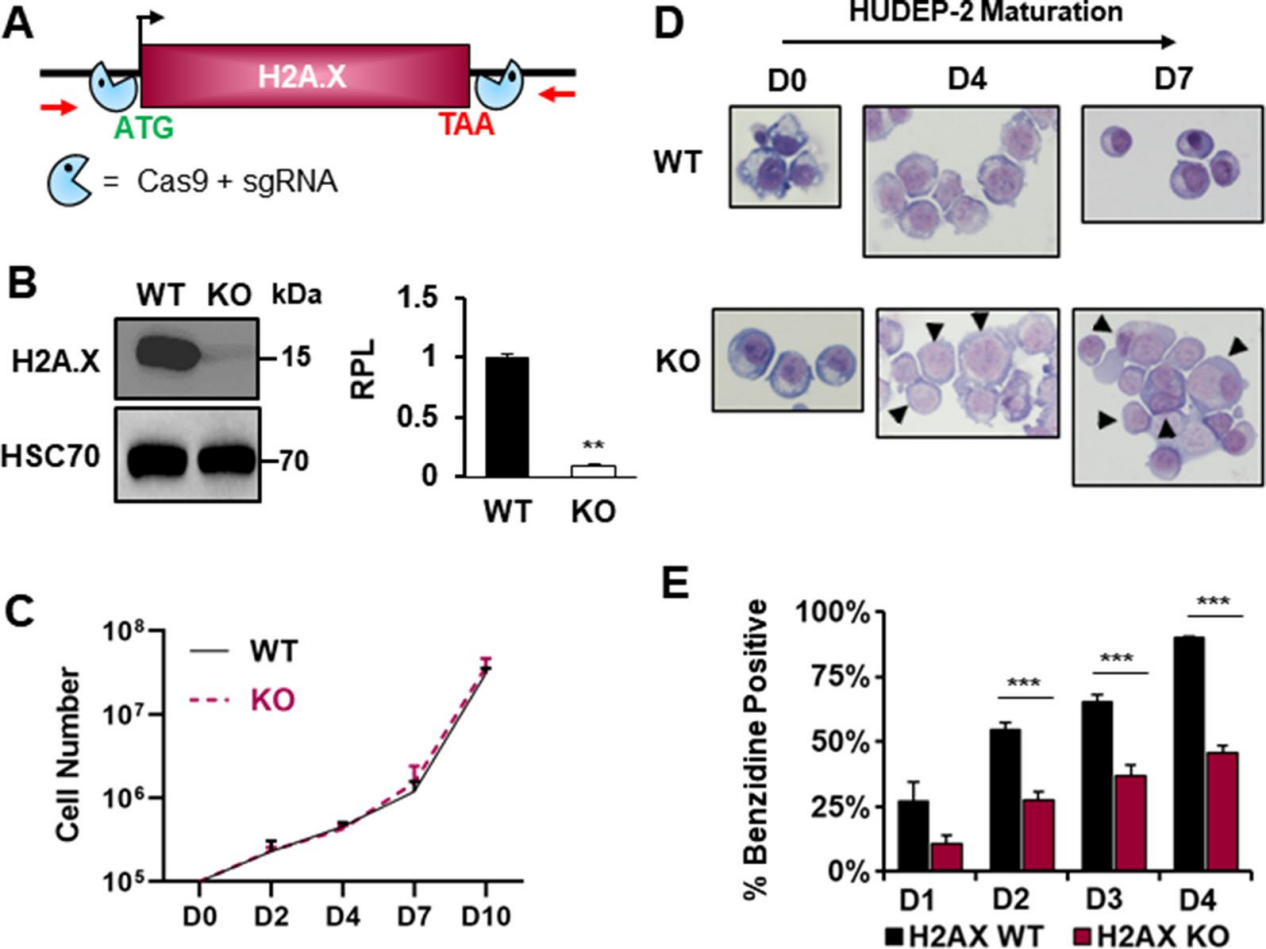
Maturation of H2A.X knockout HUDEP-2 cell lines. **A** Illustration of CRISPR/Cas9 design used to generate H2A.X KO HUDEP-2 cell lines. **B** Western blot analysis of H2A.X protein expression in WT and H2A.X KO HUDEP-2 cell lines. HSC70 was used as a loading control and quantification was performed using Image J. WT: wild type, KO: knock out. *n* = 3. **C** Graph of WT and H2A.X KO cell expansion across a HUDEP-2 maturation time course, as measured by Trypan exclusion. **D** Representative Wright–Giemsa staining of WT and H2A.X KO HUDEP-2 cells during expansion (D0), and during D4 and D7 of maturation. Arrows indicate H2A.X KO cells that have not condensed their nuclei to the same degree as WT. **E** Analysis of hemoglobin accumulation, as measured by benzidine staining, in WT and H2A.X KO HUDEP-2 cells from D1–D4 of maturation. Error bars represent ± SEM in B & E and ± SD in C. Asterisks (*) indicate significance as follows: **p ≤ 0.01; ***p ≤ 0.001. P-values were calculated with a two-tailed Student’s *t*-test

To determine the role of H2A.X in normal transcriptional dynamics associated with erythroid maturation, we generated H2A.X KOs in HUDEP-2 cells using a CRISPR/Cas9 protocol (Fig. 2A, B) [30, 31]. When grown in expansion media conditions, loss of H2A.X did not affect proliferation of HUDEP-2 cells compared to wild type (WT) (Fig. 2C). In contrast, in culture conditions that promote erythroid maturation, the loss of H2A.X resulted in cells that failed to condense their nuclei to the same degree as observed in WT cells (Fig. 2D). In addition, H2A.X KO cells did not accumulate hemoglobin during the first 4 days of maturation to the same extent as WT (Fig. 2E).

**Fig. 3:**
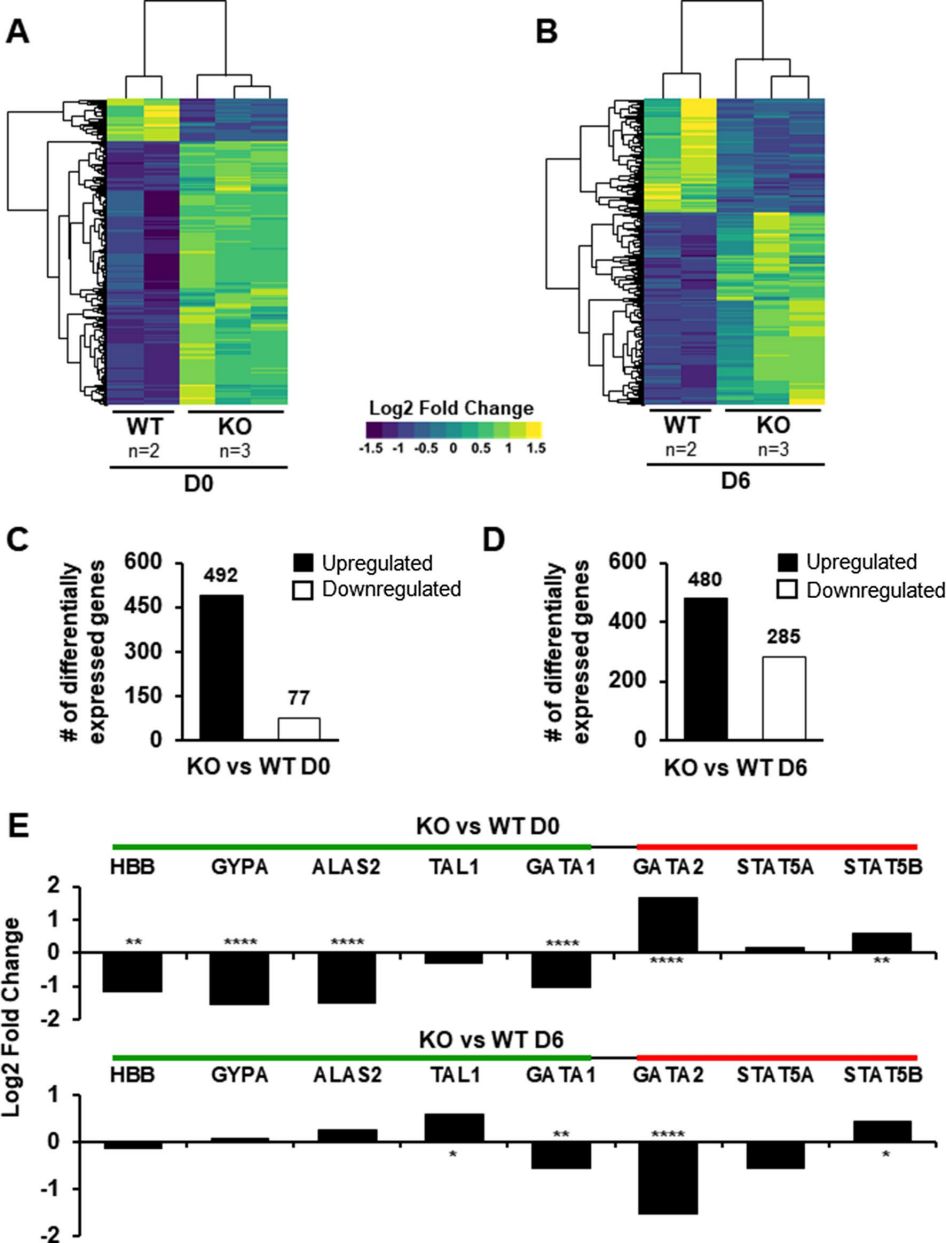
Transcriptome analysis of HUDEP-2 histone H2A.X KO cells. Heat map depicting differentially expressed genes (log2-fold change ≥ 1.5 and P-Value ≤ 0.001) in WT (n = 2) and H2A.X KO (*n* = 3) HUDEP-2 cell cultures at D0 (**A**) and D6 (**B**) of maturation. Bar graphs showing number of upregulated and downregulated genes relative to WT in H2A.X KO HUDEP-2 cell cultures at D0 (**C**) and D6 (**D**) of maturation. **E** Log2 values of fold-change for specific erythroid and hematopoietic genes at D0 and D6 of maturation. Green bar represents genes that typically are upregulated during erythropoiesis and the red bar represents genes that are typically downregulated during erythropoiesis. Asterisks (*) indicate significance as follows: *p ≤ 0.05; **p ≤ 0.01; ****p ≤ 0.0001

To further understand the effect of loss of H2A.X in erythroid cells, we performed RNA-Seq at D0 and D6 of maturation in WT and H2A.X KO HUDEP-2 subclones, and identified differentially expressed genes. At D0, 492 genes were upregulated and 77 genes downregulated in KO cells compared to WT, consistent with previous reports of a repressive role for H2A.X in the expression of subsets of genes [21]. By D6 of maturation, 480 genes were upregulated and 285 genes were downregulated (Fig. 3A–D, Additional file 1: Table S1). Gene ontology (GO) analysis on the DEGs revealed that “hemopoiesis” is populated in D0 DEGs upregulated in H2A.X KO erythroblasts, and pathways related to heme biosynthesis in D0 downregulated DEGs. By D6, however, no hematopoietic differentiation pathway was indicated by the analyses in either upregulated or downregulated DEGs in H2A.X KO erythroblasts (Additional file 2: Figure S3) [32–36].

Multiple erythroid-specific genes that are expressed at D0 and upregulated during maturation, such as β-globin (HBB), GYPA, ALAS2 and GATA1, are decreased at D0 in H2A.X KO cells, with GATA1 levels remaining lower at D6. GATA2 and STAT5b, which are typically downregulated during erythroid maturation, are significantly overexpressed in KO cells at D0 (Fig. 3E). We also observed that at D6 of maturation, the hematopoietic transcription factor TAL1 is overexpressed in KO cells. Additionally, STAT5b is overexpressed in KO cells at D6, while its expression is downregulated during normal erythroid maturation (Fig. 3E). These analyses indicate that H2A.X loss results in dysregulation of gene programs associated with late erythroid maturation, consistent with a loss of normal gene silencing programs and decreased activation of a number of late-expressed, erythroid-specific genes.

### H2A.X signals for Caspase-Initiated Chromatin Condensation

Caspase-3, which is activated by protease cleavage during apoptosis, has been shown to be necessary for normal terminal erythroid maturation [3, 8, 9, 37]. Loss of Caspase-3 results in defective nuclear maturation and enucleation [9]. However, the late stages of erythroid maturation, during which Caspase-3 activation is observed, are associated with resistance to apoptosis [10, 11]. Notably, chromatin condensation prior to cell death is a hallmark of apoptosis [38]. Given our observation that both S139 and Y142 of H2A.X are phosphorylated during late erythroid maturation, we investigated the potential link between these modifications and activation of the caspase cascade, leading to CICC.

**Fig. 4:**
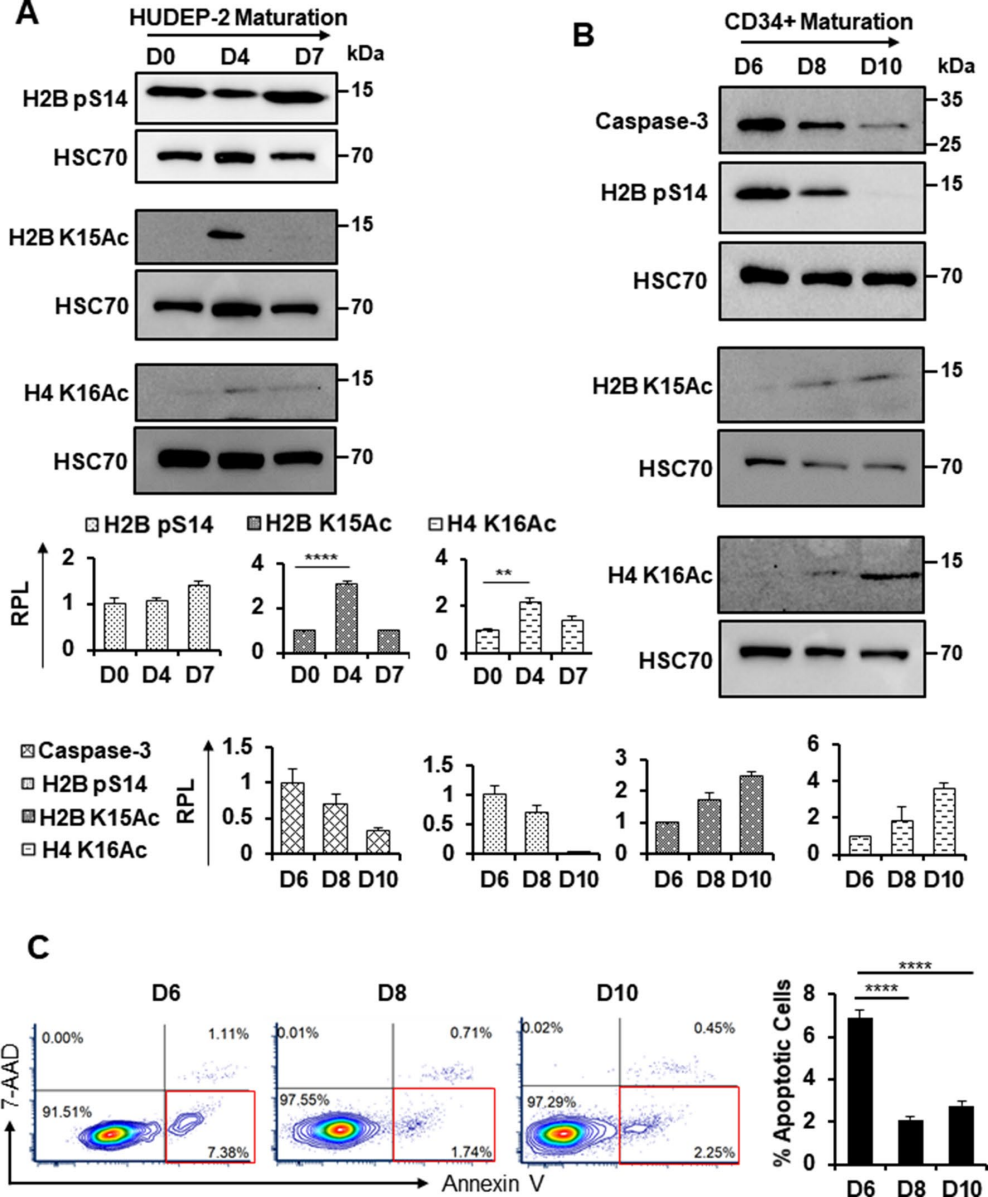
Markers of caspase-initiated chromatin condensation and apoptosis during erythroid maturation. Western blot analysis of H2B pS14, H2B K15Ac, and H4K16Ac expression during **A** HUDEP-2 (*n* = 3) and **B** human CD34+ progenitor (*n* = 2) maturation along with Caspase-3 protein expression. HSC70 was used as a loading control. Quantification was performed using ImageJ, with signal obtained at the earliest time point measured set at 1.0. p: phosphorylation, Ac: acetylation, RPL: relative protein level. **C** Schematic and graphical representations of flow cytometric analysis of Annexin-V and 7-AAD in human CD34+ progenitor cell cultures (*n* = 5 technical replicates of 2 biological replicates each). Red boxes indicate apoptotic cells. Error bars represent ± SEM. Asterisks (*) indicate significance as follows: **p ≤ 0.01; ****p ≤ 0.0001. *p*-values were calculated with a two-tailed Student’s *t*-test

In addition to H2A.X pS139 and pS142, additional post-translational marks on histones are associated with apoptosis (Additional file 2: Table S2) [39–43]. However, it is unclear whether these other chromatin modifications are also present during terminal maturation. Histone H2B S14 phosphorylation (H2B pS14), is induced by multiple factors and is associated with apoptotic chromatin condensation, and has been shown to be downstream of Caspase 3-mediated cleavage of Acinus, which is also activated during erythropoiesis [9, 39, 40]. In addition, loss of H2B K15 acetylation (H2B K15Ac) is required for H2B S14 phosphorylation to occur [39, 42]. We observed that during HUDEP-2 and human CD34+ progenitor maturation, H2B pS14 is present (Fig. 4A, B), while levels of H2B K15Ac are low or undetectable (Fig. 4A, B). These data suggest that CICC is occurring during terminal erythroid maturation.

H4 K16 acetylation (H4 K16Ac) is a histone mark that has been associated with increased chromatin accessibility subsequent to DNA damage, which can contribute to apoptosis [39, 43]. We observed that H4 K16Ac levels are also very low during HUDEP2 and CD34+ maturation (Fig. 4A, B). This suggests that during maturation, erythroid cells are not undergoing DNA damage-related cell death despite high levels of γ-H2A.X.

To further validate these findings, we looked at the amount of cell death by Annexin V staining in maturing human CD34+ progenitor cell cultures (Fig. 4C). We observed a proportion of cells (~ 7%) that stain with Annexin V at D6 of maturation and are therefore likely apoptotic, with the proportion decreasing substantially (to 1–2%) at D8 and D10 (Fig. 4C). These dynamics do not match those of H4 K16Ac, which occurs at low levels that increase from D6-D10, or H2B pS14, where a substantial decrease is only seen between D8 and D10. These differing patterns suggest that the occurrence of these marks is unrelated to apoptosis in the CD34+ cultures.

### H2A.X loss impairs the Caspase-Initiated Chromatin Condensation pathway

**Fig. 5:**
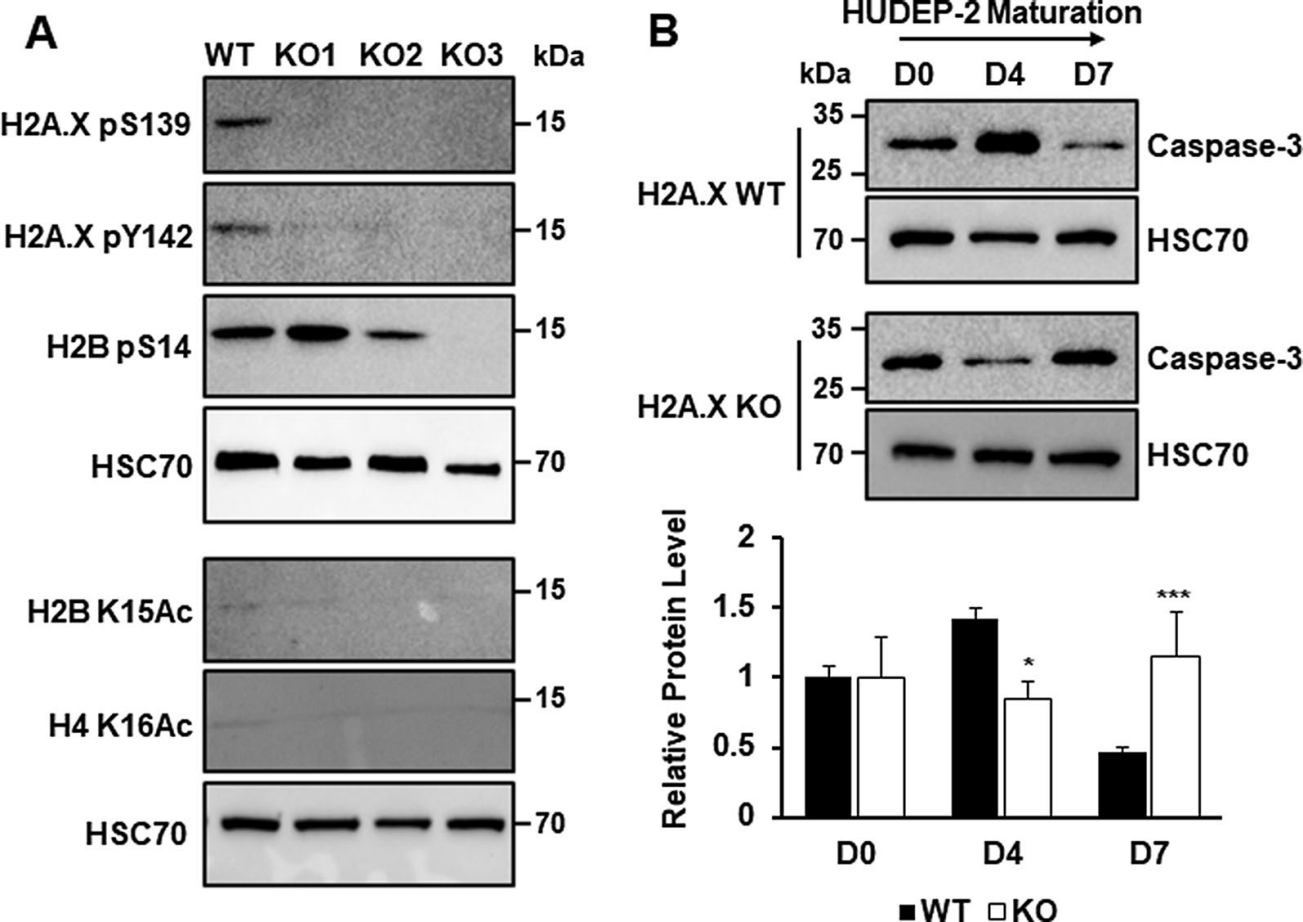
Effect of H2A.X KO on markers of caspase-initiated chromatin condensation in HUDEP-2 cells. **A** Western blot analysis of H2A.X pS139, H2A.X pY142, H2B pS14, H2B K15Ac, and H4 K16Ac post-translational marks in proliferating WT (*n* = 3) and H2A.X KO HUDEP-2 (KO1–KO3) (*n* = 2) subclones during proliferation. HSC70 was used as loading control. **B** Western blot analysis of full-length Caspase-3 protein expression in WT (*n* = 3) and H2A.X KO (*n* = 6) HUDEP-2 cell cultures at D0, D4, and D7 of maturation. HSC70 was used as loading control. Quantification was performed using ImageJ, with signal observed in WT at D0 set as 1.0. Error bars represent ± SEM. Asterisks (*) indicate significance as follows: ***p ≤ 0.001. *p*-values were calculated with a two-tailed Student’s *t*-test

Next, we assessed if CICC requires normal H2A.X function. We observed that, as expected, H2A.X pS139 and H2A.X pY142 marks are eliminated in all 3 KO lines (Fig. 5A). Furthermore, we see that H2B pS14 levels are highly variable among KO subclones, in contrast to the consistent levels exhibited by WT subclones (Fig. 5B).

Caspase-3 has been shown to be necessary for normal terminal erythroid maturation [9, 44]. During HUDEP-2 maturation, full-length Caspase-3 expression decreases by D7, most likely due to cleavage and activation of the CICC response (Fig. 5B). However, full-length Caspase-3 expression did not decrease by D7 in H2A.X KO cells, and actually increased (Fig. 5B), suggesting a loss of Caspase-3 cleavage in KO cells. This indicates that in the H2A.X KO background, the CICC pathway is not properly activated.

### BAZ1B mediates H2A.X Y142 phosphorylation and loss results in dysregulated genomic expression

**Fig. 6:**
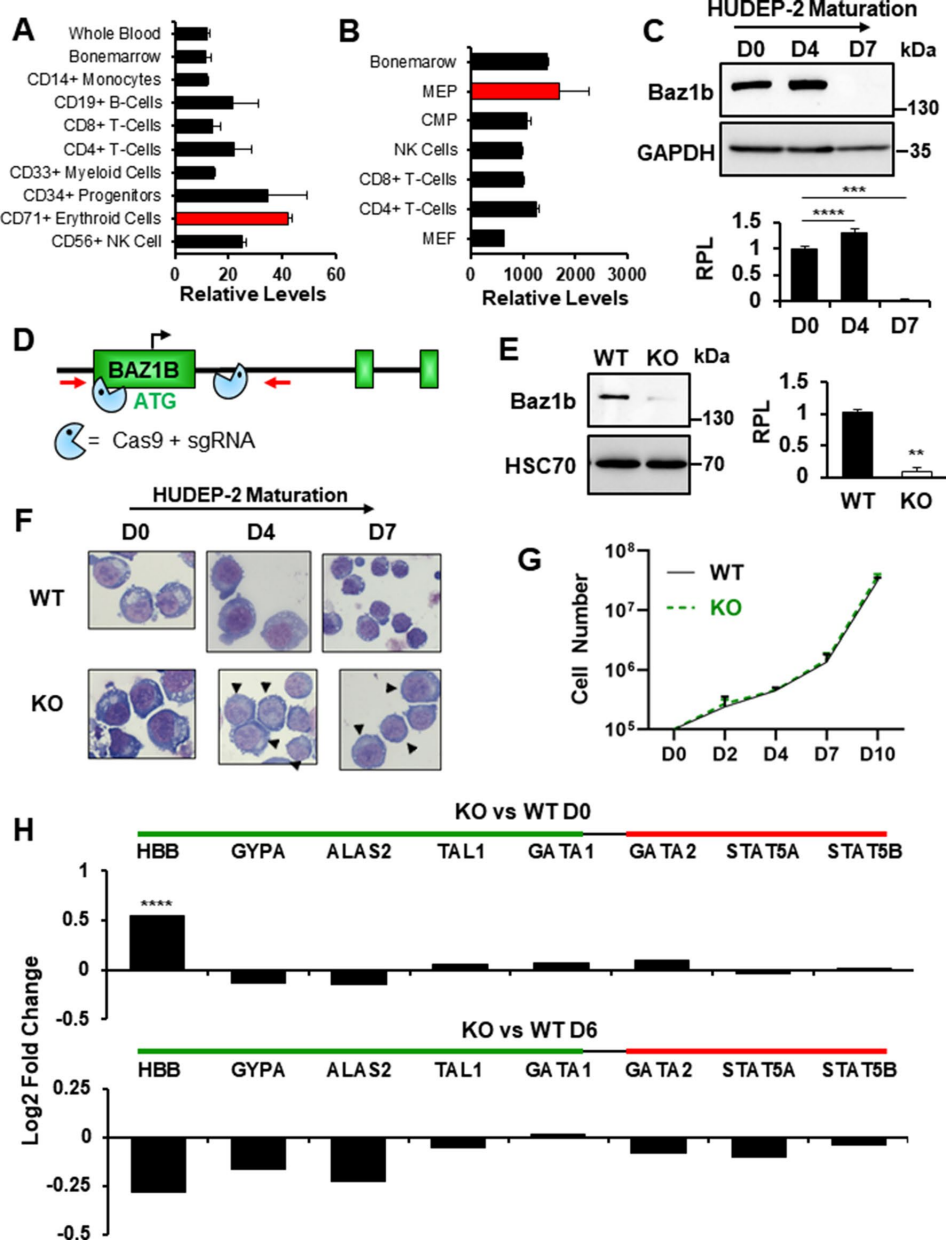
Analysis of BAZ1B knockout in HUDEP-2 cell lines. Relative mRNA expression in various cells from **A** humans and **B** mice. Data for both human and mouse mRNA expression were obtained from The Scripps Research Institute BioGPS database(46). **C** Western blot analysis of BAZ1B protein expression in WT (*n* = 3) HUDEP-2 cell cultures at D0, D4, and D7 of maturation. GAPDH was used as loading control. Quantification was performed using Image J. RPL: relative protein level. **D** Schematic of CRISPR/Cas9 designed used to generate BAZ1B KO HUDEP-2 cell lines. **E** Western blot analysis of BAZ1B protein expression in WT (*n* = 3) and BAZ1B KO (*n* = 3) HUDEP-2 cell lines. HSC70 was used as a loading control and quantification was performed using Image J. WT: wild type, KO: knock out. *p*-values were calculated with a two-tailed Student’s *t*-test. **F** Wright–Giemsa staining of WT and BAZ1B KO HUDEP-2 cells during D0, D4, and D7 of maturation. Arrows indicate BAZ1B KO cells that have not condensed their nuclei to the same degree as WT. **G** WT and BAZ1B KO cell expansion measured by Trypan exclusion. **H** Log2 values of fold-change for specific erythroid and hematopoietic genes at D0 and D6 of maturation. Green bar represents genes that typically are upregulated during erythropoiesis and the red bar represents genes that are typically downregulated during erythropoiesis. Error bars represent ± SEM in C &E and ± SD in A, B and G. Asterisks (*) indicate significance as follows: **p ≤ 0.01; ***p ≤ 0.001; ****p ≤ 0.0001

To further investigate the potential role of Y142 phosphorylation in terminal erythroid maturation and CICC, we targeted the Y142 kinase in HUDEP-2 cells. Multiple kinases phosphorylate H2A.X S139, but Y142 is only known to be phosphorylated by BAZ1B/WSTF [45]. Of note, BAZ1B mRNA is expressed at high levels in human (Fig. 6A) and mouse (Fig. 6B) erythroid cells compared to other cell types [46]. We observed robust BAZ1B protein expression at D0 of HUDEP-2 maturation, which disappears by D7 (Fig. 6C).

To test the role of the BAZ1B kinase in erythroid maturation, we utilized CRISPR/Cas9-based methods to generate BAZ1B knockout HUDEP-2 subclones (Fig. 6D, E). BAZ1B KO cells appeared phenotypically normal at D0 of maturation, and proliferated normally (Fig. 6F, G). By D7 of maturation, the BAZ1B KO cells exhibited increased cell size with large and less condensed nuclei compared to wild type (Fig. 6F). These results indicate that loss of BAZ1B leads to defective chromatin condensation during terminal erythroid maturation, similar to that observed upon loss of H2A.X.

Next, we looked for changes in gene expression in WT and BAZ1B KO HUDEP-2 cells by RNA-seq. At D0 of maturation, 27 genes were upregulated while 80 genes were downregulated in KO cells compared to WT, while at D6 of maturation, 29 genes were upregulated and 117 genes were downregulated (Additional file 2: Fig. S4a–d, Additional file 1: Table S1). Next, we performed GO analysis to determine if any pathways were specifically perturbed by the loss of BAZ1B in erythroid cells [32–36]. Pathways bearing an obvious relation to erythroid differentiation or biology did not emerge from this analysis. However, genes upregulated in the BAZ1B KO cells suggest dysregulation of nonerythroid pathways both at D0 and D6 of maturation, including cardiac terms and those related to the spinal cord. In addition, the upregulated DEGs appear to be involved in multiple metabolomic pathways (Additional file 2: Fig. S5).

In contrast to the phenotype of the H2A.X KO, we found that erythroid and hematopoietic genes are not significantly affected by the loss of BAZ1B, and exhibit nearly equivalent expression levels to their WT counterparts (Fig. 6h). In general, the H2A.X and BAZ1B KOs exhibit few differentially expressed genes in common, although this may be due to the small number of genes affected in the latter. Genes exhibiting significant changes in steady-state expression levels in BAZ1B KO cells include transcription factors involved in embryonic development and development of other tissues (SOX4 and TBX20), proteins involved in neural development, and factors involved in formation and function of the blood–brain barrier (RELN and MFSD2A) (Additional file 2: Fig. S3e). In addition, factors associated with protection against oxidative stress (SNCA and SEPN1) and genes with metabolism (CYP27B1, RYR3 and ARG2) are dysregulated (Additional file 2: Fig. S4e). These data suggest that BAZ1B-KO HUDEP-2 cells maintain the erythroid gene expression program, but exhibit changes in a small number of genes not obviously related to erythroid biology. Furthermore, the data suggest that BAZ1B is involved in nuclear condensation in maturing erythroid cells, but that this effect is largely post-transcriptional, and thus overlaps only partially with the function of H2A.X.

### Loss of BAZ1B impairs Caspase-Initiated Chromatin Condensation in erythroid cells

**Fig. 7:**
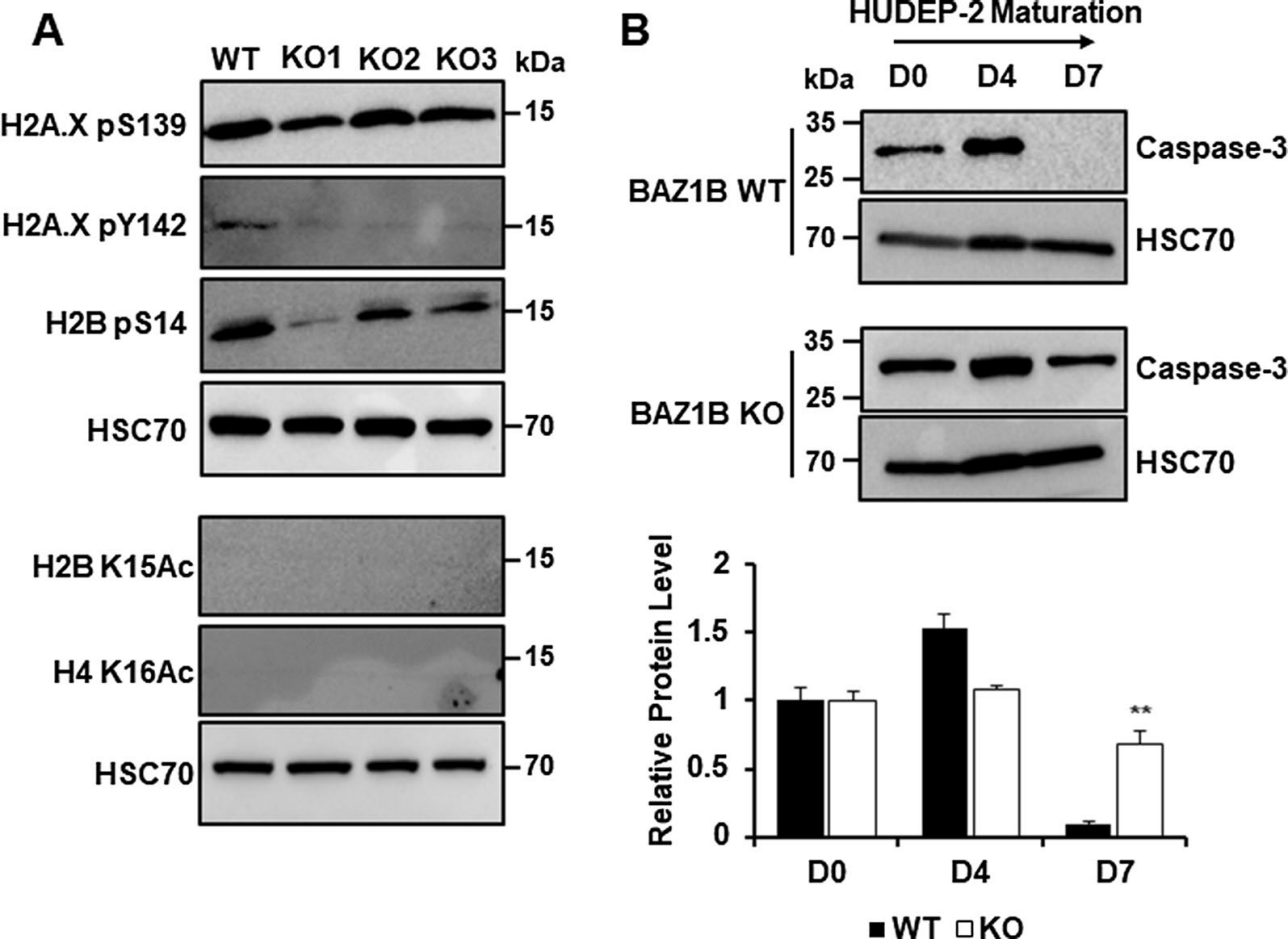
Markers of caspase-initiated chromatin condensation. **A** Western blot analysis of γ-H2A.X, H2A.X pY142, H2B pS14, H2B K15Ac, H4 K16Ac post-translational marks in WT (*n* = 3) and in BAZ1B KO HUDEP-2 subclones (KO1–KO3) (*n* = 2) during maturation. HSC70 was used as loading control. **B** Western blot analysis of full-length Caspase-3 protein expression in WT (*n* = 3) and BAZ1B KO (*n* = 6) HUDEP-2 subclones at D0, D4, and D7 of maturation. HSC70 was used as loading control. The bar graph shows quantification as performed using ImageJ. Error bars represent ± SEM. Asterisks (*) indicate significance as follows: **p ≤ 0.01. *p*-values were calculated with a two-tailed Student’s *t*-test

Next, we assessed how the loss of BAZ1B affected the post-translational marks that are associated with the CICC pathway. We observed that, as expected, H2A.X pY142 was lost in BAZ1B KO cells, but pS139 still occurred (Fig. 7A). Levels of H2B pS14 exhibit high variability between KO cell lines, but were generally decreased compared to WT, which in turn were characterized by highly consistent levels of this mark (Fig. 7A). We also observed that full-length Caspase-3 persists throughout maturation in the BAZ1B KO cells (Fig. 7B), indicating that Caspase-3 is not undergoing complete cleavage in the KO cells. These data suggest that BAZ1B phosphorylation of H2A.X Y142 is a necessary signal for the CICC pathway, and thus that loss of Y142 phosphorylation reproduces the nuclear condensation defect observed with the H2A.X KO, despite minimal effects on the transcriptome.

## Discussion

Terminal erythroid maturation is accompanied by dramatic condensation of chromatin prior to enucleation, with a selection of erythroid-specific genes expressed at high levels [1]. Prior studies have correlated histone deacetylase activity with this condensation, but beyond this correlation, the mechanisms by which chromatin compaction occurs, and the histone modifications involved, have not been elucidated [47, 48]. Here, we provide evidence that normal erythroid maturation, and associated nuclear condensation, require both histone H2A.X and the BAZ1B kinase, which affects terminal erythroid maturation.

Histone H2A.X has been extensively investigated for its role in amplifying the signal for DNA repair upon phosphorylation of S139 on its C-terminal tail (γ-H2A.X) [19, 21]. We have found that γ-H2A.X levels increase during terminal erythroid maturation, peaking at the basophilic erythroblast stage in both human and mouse. One possible explanation for this timing is that the transition to the basophilic erythroblast stage is associated with a higher proliferation rate [49] and in the environment of the transcriptionally active erythroid nucleus, this could lead to high levels of replicative stress. However, replicative stress in erythroid cells is largely alleviated by the normal function of PARP-2 [50], which is highly expressed in erythroid cells. Moreover, we found that H4 K16 acetylation, a histone mark that typically accompanies DNA damage response, occurs at low to undetectable levels in basophilic erythroblasts, and we have otherwise not found evidence for higher levels of DNA damage at this stage [39, 43]. Alternatively, a recent study has suggested that γ-H2A.X marks active replication forks; thus, high levels of γ-H2A.X could simply reflect higher replication rates and/or a higher proportion of cells in S phase [51, 52].

The γ-H2A.X signal we observe in basophilic erythroblasts appears in the context of high levels of H2A.X pY142. The kinase that phosphorylates H2A.X Y142, BAZ1B, is highly expressed in the erythroid lineage compared to other cell types [46]. These observations suggest that in late erythroid maturation, H2A.X Y142 phosphorylation is favored over dephosphorylation, and thus may persist even in the presence of H2A.X pS139. Given previous evidence that dual-phosphorylated H2A.X is recognized by pro-apoptotic factors, this suggests a potential mechanism for the initiation of apoptotic-related processes that occur in late-stage erythroblasts [5, 9, 11]. Consistent with this, loss of BAZ1B in HUDEP-2 cells eliminated phosphorylation of Y142 on histone H2A.X, and in turn resulted in inhibition of terminal maturation, correlated with loss of Caspase-3 cleavage. Notably, HUDEP-2 cells lacking either H2A.X or BAZ1B exhibited variable levels of H2B pS14. This variability was not evident among WT subclones, which were subjected to the same procedures as the KO lines, and so it is unlikely that such variability arises from extended cell culture or manipulation. Instead, we suggest that levels of this mark are normally a regulated feature of erythroid maturation downstream of histone H2A.X phosphorylation, and in the absence of this regulation are subject to unrelated, stochastic clonal variation.

Loss of histone H2A.X does not completely inhibit erythroid differentiation or prevent all nuclear condensation; H2A.X KO mice are anemic, but they are also viable [14]. Moreover, in our HUDEP-2 KO models we observed variability between clonal cell lines in the extent to which caspase cleavage was diminished. This suggests that the CICC pathway is not the sole determinant of chromatin compaction in the maturing erythroid nucleus, and/or that dual phosphorylation of histone H2A.X is supplemented by additional signals for initiation of CICC.

Comparisons of RNA-seq datasets derived from WT and BAZ1B KO HUDEP-2 cells indicated that erythroid-specific gene expression was minimally affected. This contrasts with the substantial transcriptomic changes observed upon loss of histone H2A.X, which included loss of normal expression patterns of erythroid transcription factors and other erythroid markers. Such changes are consistent with the roles this histone is known to play in maintenance of genomic integrity, chromatin condensation and silencing of specific gene expression programs [15–19]. We therefore suggest that BAZ1B, and Y142 phosphorylation, are specifically involved in post-translational events leading to caspase activation and CICC. Histone H2A.X, however, has a more expansive function in modulating gene expression patterns relevant to erythroid maturation in addition to nuclear condensation. Our results thus indicate that histone H2A.X serves multiple roles in late-stage erythroid maturation.

**Fig. 8:**
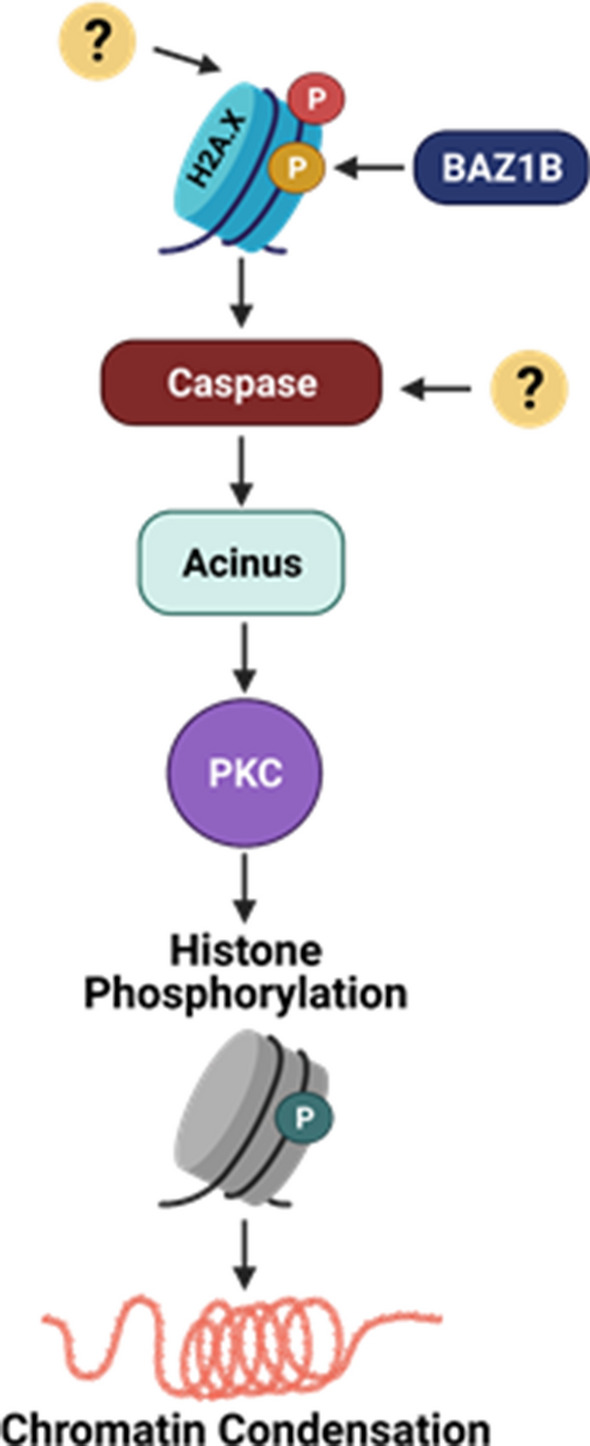
Caspase-initiated chromatin condensation pathway. Model representing the predicted pathway that erythroblasts undergo to condense their chromatin. Question marks indicate uncertainty regarding the initial signal for H2A.X S139 phosphorylation at the basophilic erythroblast stage, and the possibility of additional, redundant signals for caspase activation. (p) in red circle represents γ-H2A.X; (p) in yellow circle represents H2A.X pY142; (p) in blue circle represents H2B pS14. The illustration was created using bioRENDER

Our results, in combination with multiple prior studies, suggest a more detailed (if speculative) model for how select aspects of apoptotic pathways are induced in the context of erythroid maturation (Fig. 8). The requirement for Caspase 3 activity in erythroid maturation has previously been demonstrated, and this activity is known to lead to cleavage of Caspase 3 substrates in erythroid precursors, including the apoptotic chromatin condensation factor Acinus [3, 8, 9, 44]. Studies in other systems have shown that Acinus in turn activates kinases that phosphorylate histone H2B S14 [40, 42]. Our observations suggest a potential initiating signal for the caspase cascade at a specific stage of erythropoiesis (BasoE), and demonstrate that loss of either histone H2A.X or the BAZ1B kinase disrupts this pathway, including the phosphorylation of H2B S14. These findings are consistent with the suggestion that terminal erythroid maturation may represent a “special form of apoptosis” [11]. Notably, however, there may be other initiating signals, and histone H2A.X itself appears to have an additional role in erythroid maturation than that served by its post-translational modifications alone.

## Materials and methods

### Mice and tissues

H2A.X heterozygous mice were generously provided by Dr. Andre Nussenzweig (National Cancer Institute, NIH, USA). C57BL/6 (WT) and H2A.X KO mice were obtained through mating of H2A.X heterozygous mice; Mendelian ratios were observed in progeny. Bone marrow was obtained from femurs of H2A.X KO, C57BL/6, and CD1 (Charles River) mice as previously described [53] and flushed through a 40-mm cell strainer (BD Biosciences) for flow cytometry-based assays. Peripheral blood was collected from H2A.X KO and C57BL/6 mice from the submandibular vein, and standard blood parameters were measured using a HemaTrue Veterinary Hematology Analyzer (HESKA Corp.) and by flow analysis on an LSR-II flow cytometer (BD Biosciences). All animal experiments were approved by the University of Rochester Committee on Animal Resources (UCAR) and performed in accordance with the National Institutes of Health Guide for the Care and Use of Laboratory Animals.

### Cell culture

HUDEP-2 cells were maintained in StemSpan SFEM containing 50 ng/mL human SCF (PeproTech), 3 U/mL erythropoietin, 2 mM dexamethasone (Sigma), and 1 mg/mL doxycycline (Sigma). Cells were differentiated for 7 days in Iscove’s Modified Dulbecco’s Medium (IMDM) containing 5% PB plasma (STEMCELL Technologies), 2 U/mL heparin (Sigma), 1% GlutaMAX (Gibco), 330 mg/mL holo-transferrin (Sigma), 100 ng/mL human SCF, 10 mg/mL insulin (Tocris Bioscience), 3 U/mL erythropoietin, and 1 mg/mL doxycycline. MEL-745A cells were cultured in Dulbecco Modified Eagle Medium (DMEM) containing 10% FBS (Gemini Bio), 1% GlutaMAX, and 1% penicillin/streptomycin. For differentiation, cells were cultured for 4 days in culture medium with 2% dimethyl sulfoxide (DMSO) (Fisher).

CD34^+^ cells were thawed and first cultured in StemSpan H3000 (STEMCELL Technologies) and StemSpan CC100 (STEMCELL Technologies) for 3 days. They were then subjected to CD36^+^ selection and differentiated as previously described [29], with cells harvested at D6, D8, and D10 of maturation.

### CRISPR/Cas9-mediated genomic deletions

For targeted genes, sgRNAs were designed to regions located upstream of the start codon and either just downstream of the stop codon (histone H2A.X) or downstream of the first exon (BAZ1B/WSTF) following a previously described protocol [30, 31] (Additional file 2: Table S3). sgRNA sequences were subcloned into LentiCRISPRv2GFP and LentiCRISPRv2-mCherry plasmids. HUDEP-2 cells were transduced by spinoculation for 1.5 h at 37 °C and then placed overnight in maintenance medium with viral supernatant. The next day, the cells were pelleted to remove viral supernatant and transferred to fresh maintenance media. At 48 h post-transduction, the cells were stained with 7AAD (BioLegend) and sorted (BioRad S3e) for live, GFP+ and mCherry+ populations. 7 days after the sort, the cells were plated into a 96-well plate at 1 cell/100 µL (e.g., an average of 1 cell/well). A week after dilution, colonies were subjected to gDNA prep and homozygous deletions were identified by PCR of the surrounding regions (Additional file 2: Table S2) and then Sanger sequenced for confirmation. LentiCRISPRv2GFP was a gift from David Feldser (Addgene plasmid # 82416; http://n2t.net/addgene:82416; RRID: Addgene_82416). LentiCRISPRv2-mCherry was a gift from Agata Smogorzewska (Addgene plasmid # 99154; http://n2t.net/addgene:99154; RRID: Addgene_99154).

### Antibodies and reagents

See Additional file 2: Table S4 for list of antibodies and reagents used.

### Protein extraction and western blotting

SDS lysis buffer (50 mM Tris pH 8.0, 10 mM EDTA, 1% SDS, 1X Proteinase inhibitor (Roche), PMSF, and DTT) and sonication (Bioruptor UCD-200, Diagenode) was used to extract total protein from roughly ~ 1 × 10^6^ HUDEP-2, MEL, and primary CD34+ cultured cells. Protein visualization was performed through electrophoresis by loading roughly 10^5^ cell equivalents/well onto 10–20% Tris–glycine gels (ThermoFisher). Gels were then transferred to 0.2 mm nitrocellulose blotting membrane (GE Life Sciences) and probed with specific antibodies. Quantification was performed using ImageJ and loading control was used for normalization. Either HSC70 or GAPDH were used as loading control based on which gave the best separation between bands. Lack of asterisks indicate not significant. Stats were not calculated for primary cultures due to only being an *n* = 2. See Additional file 2: Table S4 for antibodies used.

### RNA-Seq and bioinformatic analysis

HUDEP-2 cells (WT and H2A.X- and BAZ1B KO) were taken at D0 and D6 of maturation and RNA was extracted using the RNeasy Plus Kit (Qiagen) following manufacturer’s instructions. Total RNA concentration was determined using the NanoDrop 1000 spectrophotometer and RNA quality was assessed with the Agilent Bioanalyzer. TruSeq Stranded mRNA Sample Preparation Kit (Illumina) was used for next generation sequencing library construction per manufacturer’s protocols. The amplified libraries were then hybridized to the Illumina Single end flow cell and amplified using the NextSeq550 DNA Sequencer (Illumina). Single end reads of 75 nt were generated for each sample. Raw reads were generated from Illumina HiSeq2500 sequencing and demultiplexed using bcl2fastq version 2.19.0. Quality filtering and adaptor removal was performed using fasp 0.20.0. Processed and cleaned reads were then mapped to the human reference genome (GRCh39/hg38 and gencode31) using STAR version 2.7.0. Differential expression analyses and data normalization was performed using DESeq2 version 1.22.1 with an adjusted p-value threshold of 0.05 within the R environment version 3.5.1. DEGs were defined by a greater than 1.5 log twofold change (or 2.8-fold) and *p*-value < 0.001. See Additional file 1: Table S1 for DEGs.

### γ-H2A.X quantification

All quantification was performed in IDEAS 4.0 following analysis on the ImageStream platform. Erythroid precursor populations (CFU-E, ProE, BasoE, PolyE, OrthoE) were gated as described previously [25, 27]. To quantify γ-H2A.X nuclear staining, a custom mask was created that combined a nuclear morphology mask with a minimum γ-H2A.X intensity of 100 with a spot mask (5:1 spot to background ratio with radius of 1). Spot count function was then utilized with this custom mask to specifically quantify the number of γ-H2A.X+ spots within individual erythroblasts.

### Apoptosis quantification

Human CD34+ progenitor cells (D6–D10) were stained with Annexin V-FITC and 7-AAD for 20 min and then analyzed on the LSR-II platform (BD Biosciences) with isotype controls. All quantification and analysis were performed using FCS Express 7 (De Novo Software). See Additional file 2: Table S4 for reagent information.

## Supplementary Information

**Additional file 1.** Differentially expressed genes (DEGs) derived from RNA-seq analyses of wild-type (wt) vs. H2A.X or BAZ1B knockout HUDEP-2 cell lines.  Contains lists of DEGs (log base 2 +/−1.5 change) identified by comparison of wt vs. H2A.X KO lines, or of wt vs. BAZ1B KO lines, at d0 and d6 of maturation.

**Additional file 2.** Additional Figures and Tables.

## Data Availability

RNA-Seq data sets are available at NCBI GEO GSE GSE159422.
