# Supplementary material for: Histone H2A.X phosphorylation and Caspase-Initiated Chromatin Condensation in late-stage erythropoiesis
Source: Epigenetics Chromatin. 2021 Jul 30;14:37. doi: 10.1186/s13072-021-00408-5 (PMC8325214; doi:10.1186/s13072-021-00408-5)
Supplement: Supplementary file 2 — Additional file 2. Additional Figures and Tables. [file 13072_2021_408_MOESM2_ESM.pdf]

Figure S1

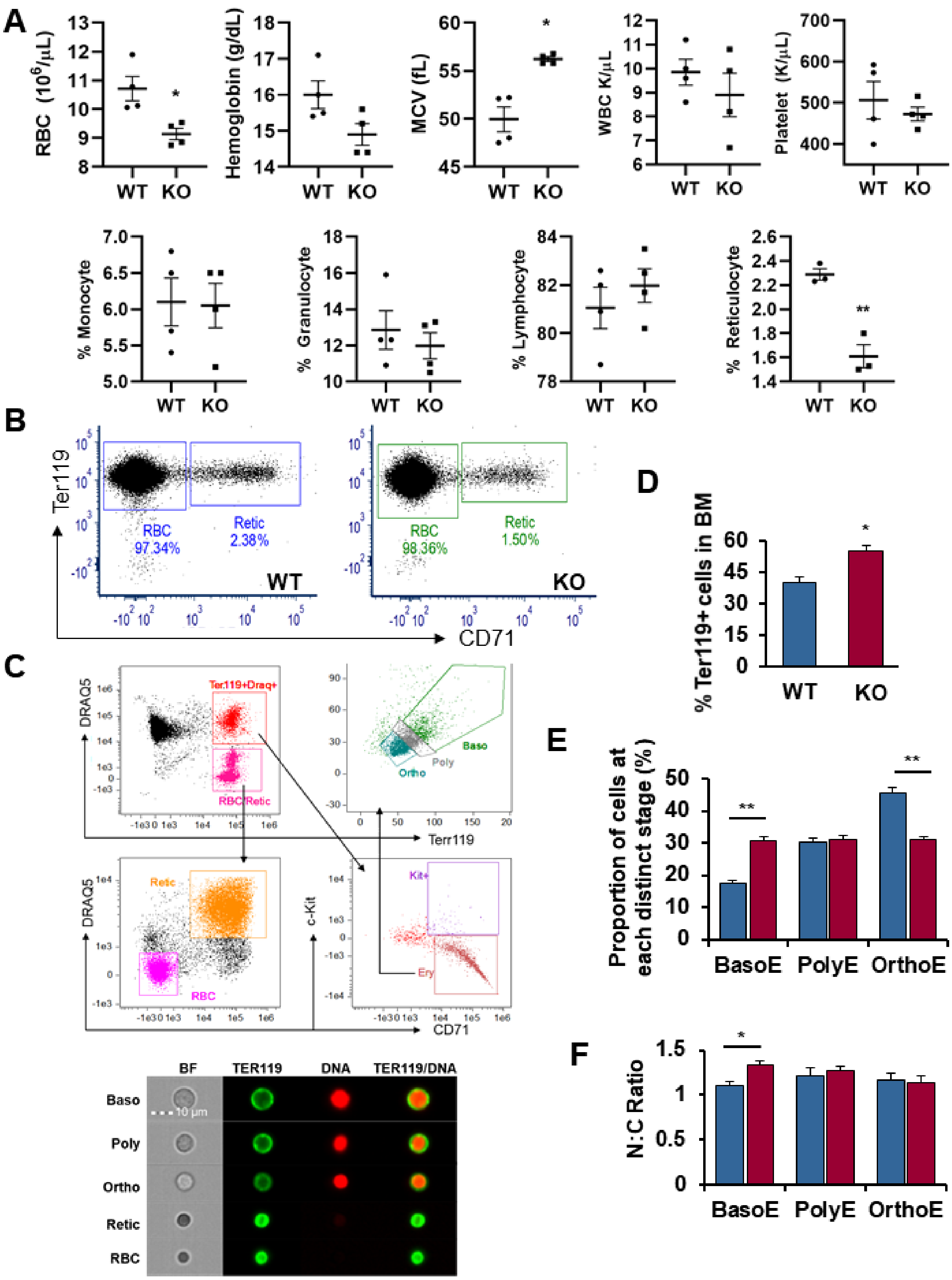

A
RBC (10^6/μL)
Hemoglobin (g/dL)
MCV (fL)
WBC K/μL
Platelet (K/μL)
% Monocyte
% Granulocyte
% Lymphocyte
% Reticulocyte
WT
KO
B
Ter119
CD71
RBC
97.34%
Retic
2.38%
WT
RBC
98.36%
Retic
1.50%
KO
C
DRAQ5
Ter119+Draq+
RBC/Retic
Terr119
Baso
Poly
Ortho
Retic
RBC
c-Kit
Kit+
Ery
CD71
BF
TER119
DNA
TER119/DNA
Baso
Poly
Ortho
Retic
RBC
10 μm
D
% Ter119+ cells in BM
E
Proportion of cells at each distinct stage (%)
BasoE
PolyE
OrthoE
F
N:C Ratio

Figure S2

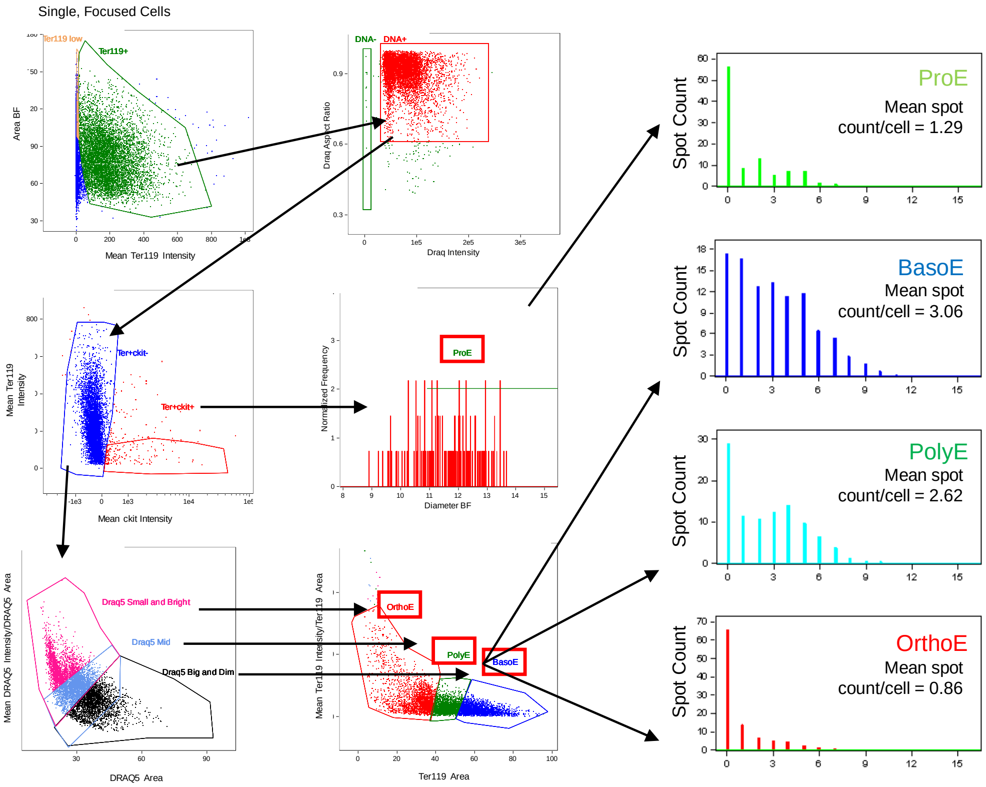

Single, Focused Cells
Ter119 low
Ter119+
Area BF
Mean Ter119 Intensity
DNA-
DNA+
Draq Aspect Ratio
Draq Intensity
Ter+ckit-
Ter+ckit+
Mean Ter119 Intensity
Mean ckit Intensity
ProE
Normalized Frequency
Diameter BF
Draq5 Small and Bright
Draq5 Mid
Draq5 Big and Dim
Mean DRAQ5 Intensity/DRAQ5 Area
DRAQ5 Area
OrthoE
PolyE
BasoE
Mean Ter119 Intensity/Ter119 Area
Ter119 Area
Spot Count
ProE
Mean spot count/cell = 1.29
BasoE
Mean spot count/cell = 3.06
PolyE
Mean spot count/cell = 2.62
OrthoE
Mean spot count/cell = 0.86

Figure S3

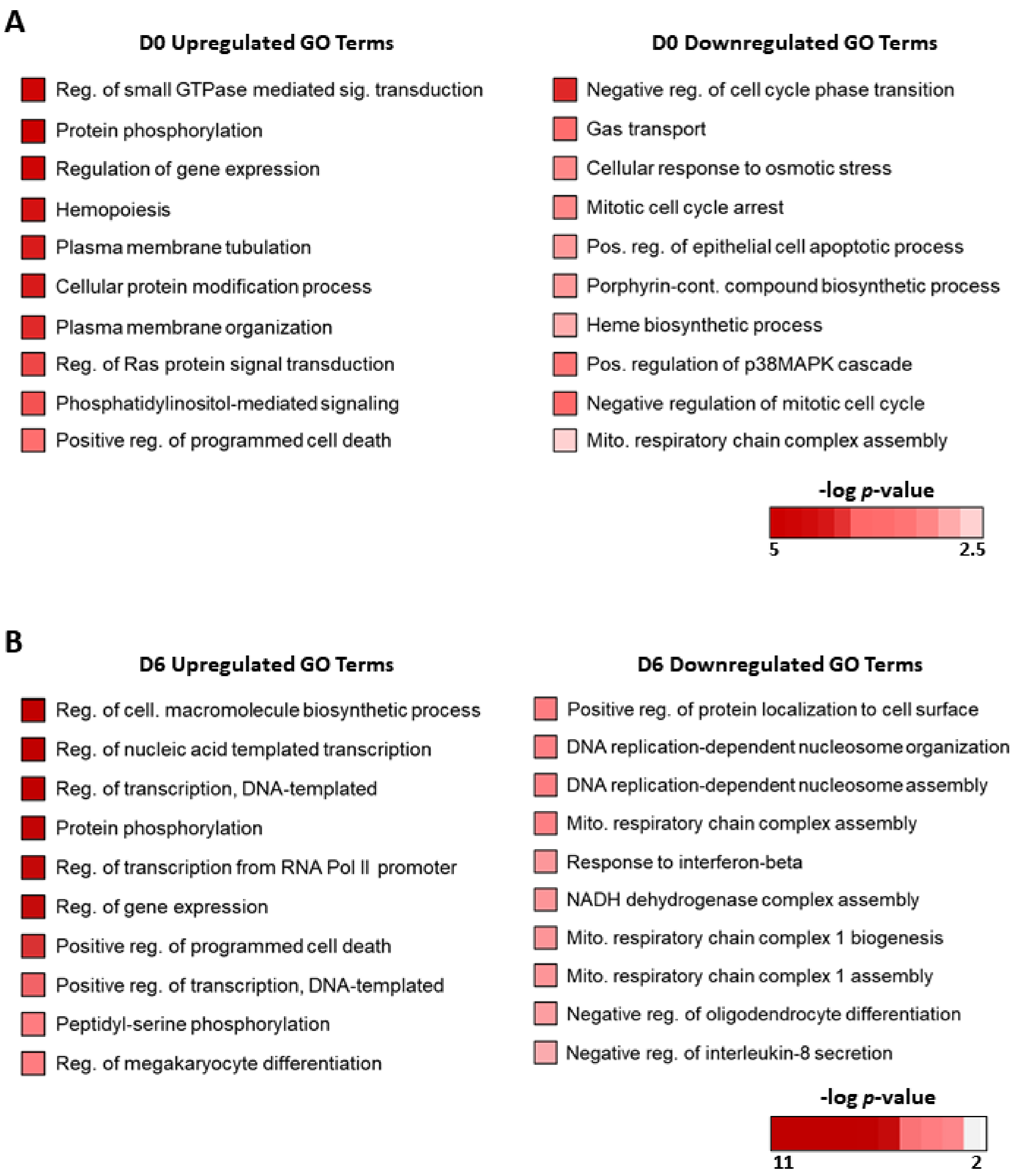
A
D0 Upregulated GO Terms
Reg. of small GTPase mediated sig. transduction
Protein phosphorylation
Regulation of gene expression
Hemopoiesis
Plasma membrane tubulation
Cellular protein modification process
Plasma membrane organization
Reg. of Ras protein signal transduction
Phosphatidylinositol-mediated signaling
Positive reg. of programmed cell death
D0 Downregulated GO Terms
Negative reg. of cell cycle phase transition
Gas transport
Cellular response to osmotic stress
Mitotic cell cycle arrest
Pos. reg. of epithelial cell apoptotic process
Porphyrin-cont. compound biosynthetic process
Heme biosynthetic process
Pos. regulation of p38MAPK cascade
Negative regulation of mitotic cell cycle
Mito. respiratory chain complex assembly
-log *p*-value
5
2.5
B
D6 Upregulated GO Terms
Reg. of cell. macromolecule biosynthetic process
Reg. of nucleic acid templated transcription
Reg. of transcription, DNA-templated
Protein phosphorylation
Reg. of transcription from RNA Pol II promoter
Reg. of gene expression
Positive reg. of programmed cell death
Positive reg. of transcription, DNA-templated
Peptidyl-serine phosphorylation
Reg. of megakaryocyte differentiation
D6 Downregulated GO Terms
Positive reg. of protein localization to cell surface
DNA replication-dependent nucleosome organization
DNA replication-dependent nucleosome assembly
Mito. respiratory chain complex assembly
Response to interferon-beta
NADH dehydrogenase complex assembly
Mito. respiratory chain complex 1 biogenesis
Mito. respiratory chain complex 1 assembly
Negative reg. of oligodendrocyte differentiation
Negative reg. of interleukin-8 secretion
-log *p*-value
11
2

Figure S4

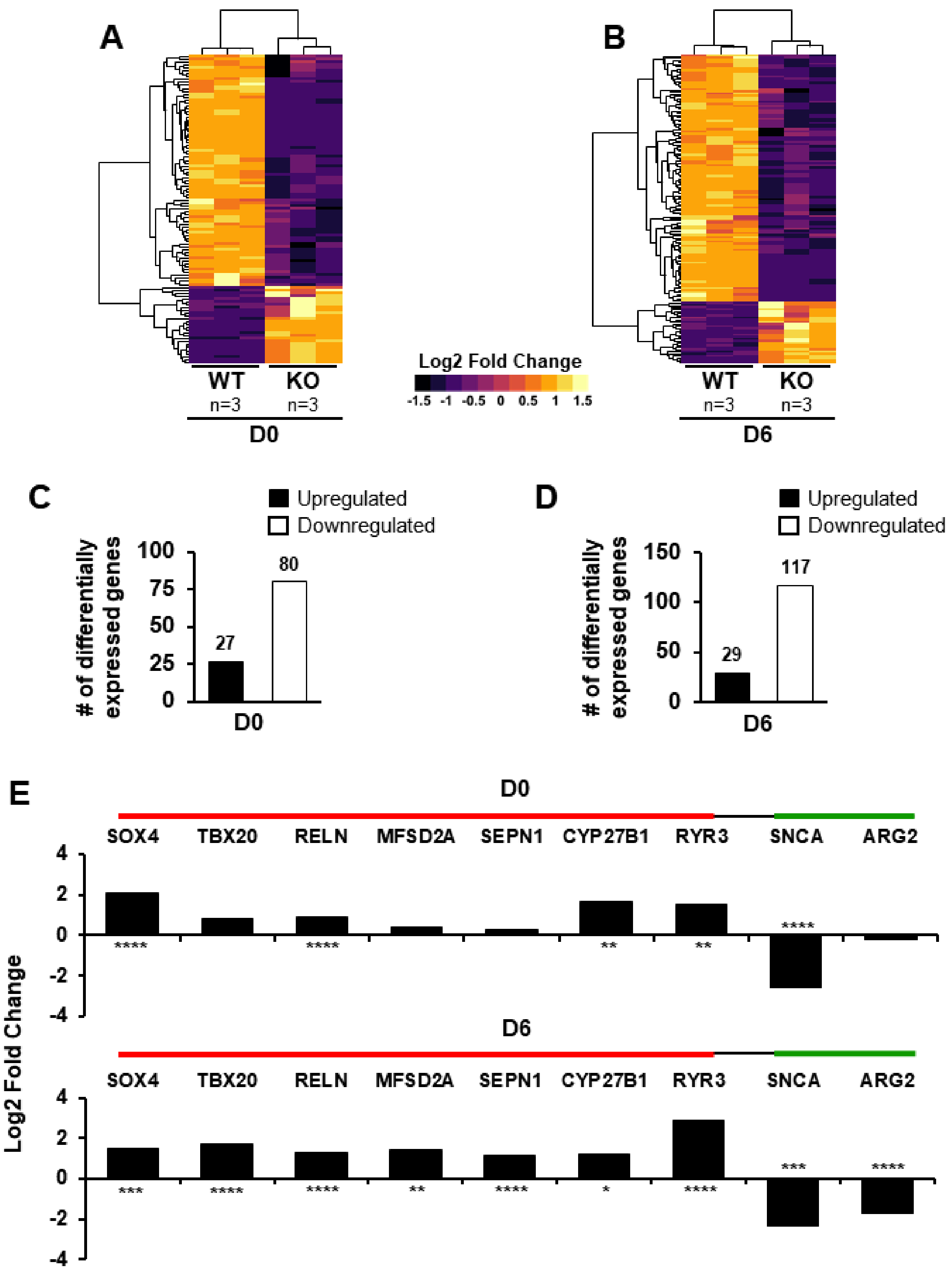
A
WT
n=3
KO
n=3
D0
B
WT
n=3
KO
n=3
D6
Log2 Fold Change
-1.5 -1 -0.5 0 0.5 1 1.5
C
Upregulated
Downregulated
# of differentially expressed genes
100
75
50
25
0
27
80
D0
D
Upregulated
Downregulated
# of differentially expressed genes
150
100
50
0
29
117
D6
E
D0
SOX4 TBX20 RELN MFSD2A SEPN1 CYP27B1 RYR3 SNCA ARG2
4
2
0
-2
-4
**** **** ** ** ****
D6
SOX4 TBX20 RELN MFSD2A SEPN1 CYP27B1 RYR3 SNCA ARG2
4
2
0
-2
-4
*** **** **** ** **** * **** *** ****
Log2 Fold Change

Figure S5

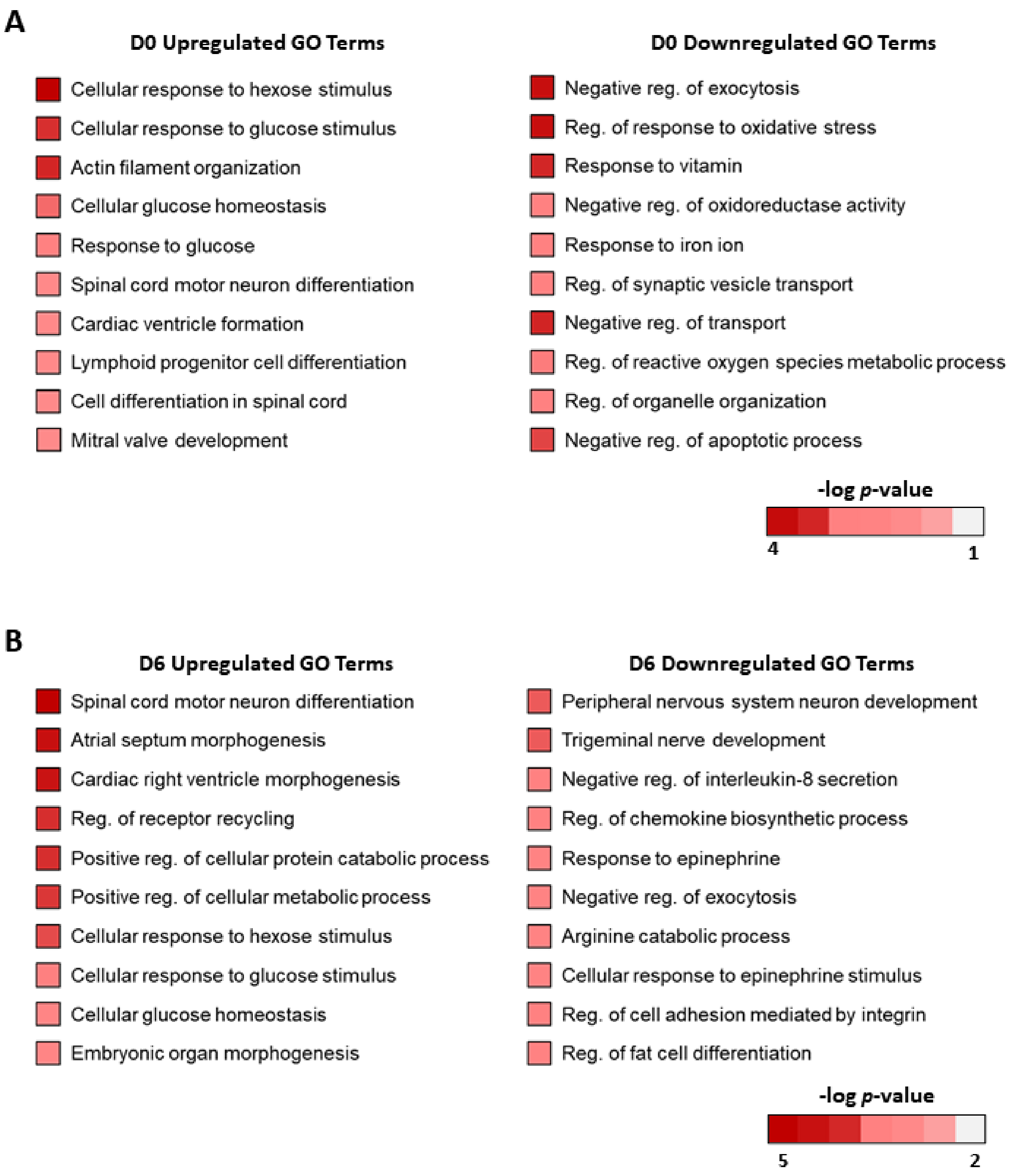
A
D0 Upregulated GO Terms
Cellular response to hexose stimulus
Cellular response to glucose stimulus
Actin filament organization
Cellular glucose homeostasis
Response to glucose
Spinal cord motor neuron differentiation
Cardiac ventricle formation
Lymphoid progenitor cell differentiation
Cell differentiation in spinal cord
Mitral valve development
D0 Downregulated GO Terms
Negative reg. of exocytosis
Reg. of response to oxidative stress
Response to vitamin
Negative reg. of oxidoreductase activity
Response to iron ion
Reg. of synaptic vesicle transport
Negative reg. of transport
Reg. of reactive oxygen species metabolic process
Reg. of organelle organization
Negative reg. of apoptotic process
-log p-value
4
1
B
D6 Upregulated GO Terms
Spinal cord motor neuron differentiation
Atrial septum morphogenesis
Cardiac right ventricle morphogenesis
Reg. of receptor recycling
Positive reg. of cellular protein catabolic process
Positive reg. of cellular metabolic process
Cellular response to hexose stimulus
Cellular response to glucose stimulus
Cellular glucose homeostasis
Embryonic organ morphogenesis
D6 Downregulated GO Terms
Peripheral nervous system neuron development
Trigeminal nerve development
Negative reg. of interleukin-8 secretion
Reg. of chemokine biosynthetic process
Response to epinephrine
Negative reg. of exocytosis
Arginine catabolic process
Cellular response to epinephrine stimulus
Reg. of cell adhesion mediated by integrin
Reg. of fat cell differentiation
-log p-value
5
2

Supplemental Figure Legends

Figure S1: Characterization of blood parameters and late erythroid maturation in H2A.X-null mice. (A) Complete blood counts performed on WT (C57BL/6) and H2A.X-null mice. Each dot is a single mouse (n = 4 for all except for reticulocyte count n = 3). RBC = red blood cell count; MCV = mean cell volume; WBC = white blood cell count. **(B)** Schematic of flow cytometric analysis of CD71 and Ter119 levels in murine peripheral blood from WT and H2A.X-null mice to obtain reticulocyte count performed on FCS Express 7**.** Quantification was performed using IDEAS 4.0 following analysis on the ImageStream platform. **(C)** Flow cytometric analysis of WT (n = 3) and H2A.X KO (n = 3) murine bone marrow, gated as previously described[25,50], and resulting images of different stages of erythropoiesis obtained through gating. **(D)** Percentage of Ter119 positive cells in WT and H2A.X-null murine bone marrow. **(E)** Bar graphs of imaging flow cytometric analysis of the erythroid population utilizing Ter119 and DRAQ staining. **(F)** N:C ratio of erythroid cells of the indicated maturational stages. N:C ratio was calculated by dividing nuclear area of a cell by its cellular area. N:C for each cell then was averaged and graphed. Error bars represent standard error of the mean. Error bars represent ± SEM. Asterisks (*) indicate significance as follows: *$p \leq 0.05$; **$p \leq 0.01$.

Figure S2: Gating and classification of erythroid cells and quantitation of γ-H2A.X foci. (A) Quantitation was performed using IDEAS 4.0 following analysis on the ImageStream platform. Erythroid precursor populations (CFU-E, ProE, BasoE, PolyE, OrthoE) were gated as described previously[25,50] and as above. To quantify γ-H2A.X

nuclear staining, a custom mask was created that combined a nuclear morphology mask with a minimum γ -H2A.X intensity of 100 with a spot mask (5:1 spot to background ratio with radius of 1). Spot count function was then utilized with this custom mask to gspecifically quantify the number of γ -H2A.X+ spots within individual erythroblasts.

Figure S3: H2A.X GO Terms for DEGs at D0 and D6 of maturation.

Gene ontology analysis was performed using Enrichr on both upregulated and downregulated differentially expressed genes (log2-fold change >1.5, and P-Value <0.001) at **(A)** D0 and **(B)** D6. Color scale indicates the -log(*p*-value) of the *p*-value that Enrichr gave each term. Pos. = Positive, Reg. = Regulation, cell. = cellular, Mito. = Mitochondria.

Figure S4: Transcriptome Analysis of WT and BAZ1B KO HUDEP-2 Cell cultures.

Heat map depicting differentially expressed genes (log2-fold change >1.5 and P-Value <0.001) between WT (n = 3) and BAZ1B KO (n = 3) HUDEP-2 cell cultures at D0 **(A)** and D6 **(B)** of maturation. Bar graphs of the number of upregulated and downregulated genes in BAZ1B KO HUDEP-2 cell cultures at D0 **(C)** and D6 **(D)** of maturation. **(E)** Log2 values of fold-change for specific genes at D0 and D6 of maturation. Green bar represents genes that typically are upregulated during erythropoiesis and the red bar represents genes that are typically downregulated during erythropoiesis. Asterisks (*) represent significance as follows: * $p \leq 0.05$; **$p \leq 0.01$; ***$p \leq 0.001$; ****$p \leq 0.0001$.

Figure S5: BAZ1B GO Terms for DEGs at D0 and D6 of maturation.

Gene ontology analysis was performed using Enrichr on both upregulated and downregulated differentially expressed genes (log2-fold change >1.5, and P-Value <0.001) at **(A)** D0 and **(B)** D6. Color scale indicates the -log(*p*-value) of the *p*-value that Enrichr gave each term. Pos. = Positive, Reg. = Regulation, cell. = cellular, Mito. = Mitochondria

Table S2: post-translational marks associated with apoptosis

Histone PTMs	Function
H2A.X pS139	**+:** Signals for either DNA repair or apoptosis by recruiting either pro-repair or pro-apoptotic factors
H2A.X pY142	**+:** Recruits pro-apoptotic factors and inhibits recruitment of repair factors<br>**-:** Loss results in repair factors being recruited due to presence of γ-H2A.X
H2B pS14	**+:** Promotes chromatin condensation; signal can be enhanced through Acinus activated PKC-δ activation
H2B K15Ac	**+:** Present on non-dying cells<br>-: Loss of this mark is required for H2B S14 phosphorylation
H4 K16Ac	**+:** Promotes DNA damage induced cell death<br>**-:** Loss or lack of this mark reduces DNA damage induced cell death

Table S3: CRISPR/Cas9 Primers

Primer	Sequence (5' to 3')	Notes
H2AX_5-Out_Fwd	GATGTCGGGCCGCGGCAAGAC	Targets before start codon
H2AX_5-Out_Rev	GTCTTGCCGCGGCCCGACATC	
H2AX_3-Out_Fwd	GCTTGCCCCGCAGTCTGAAG	Targets after stop codon
H2AX_3-Out_Rev	CTTCAGACTGCGGGGCAAGC	
H2AX_Screen_Fwd	GTT AAC CGC AAC CAA CCG	WT amplicon size: 1053bp<br>KO amplicon size: ~500bp
H2AX_Screen_Rev	GCC AAG TCT TCC AGA AGG TGC	
BAZ1B_5-Out_Fwd	GCC ATC GCG GCG GCG GCG GTG	Targets before start codon
BAZ1B_5-Out_Rev	CAC CGC CGC CGC CGC GAT GGC	
BAZ1B_3-Out_Fwd	GCC TTG AGG ACC CGA GAG GG	Targets intron between exon 1 and 2
BAZ1B_3-Out_Rev	CCC TCT CGG GTC CTC AAG GC	
BAZ1B_Screen_Fwd	CTG CTG AGG AGG AGT CGT G	WT amplicon size: 1027bp<br>KO amplicon size: ~565bp
BAZ1B_Screen_Rev	CCA CAG GCA GTTTCCTTC	

Table S4: Antibodies & Reagents

Antibody	Company	Catalogue Number
Histone H2A.X	Abcam	Ab20669
H2A.X pS139	Cell Signaling	9718
H2A.X pY142	Thermo Fisher Scientific	PA5-40153
H2B pS14	Cell Signaling	6959
H2B K15Ac	Cell Signaling	9083
Histone H3	Cell Signaling	4499
H4 K16Ac	Abcam	Ab109463
Caspase-3	Cell Signaling	14220
BAZ1B/WSTF	Abcam	Ab51256
HSC70	Santa Cruz	SC7298
GAPDH	Cell Signaling	2118
CD117-Pacific Blue	BioLegend	105819
CD71-PE	BioLegend	113807
Ter119-AF488	BioLegend	116215
Ter119-PE	BD BioScience	553673
Ter119-PE/Cy7	BioLegend	116215
Annexin-V-FITC	BioLegend	640905
GR1-PerCP/Cy5.5	BioLegend	108427
Hoechest 33342	Invitrogen	H3570
DRAQ5	eBioscience	65-0880-92
7-AAD	BioLegend	420404
Annexin V Binding Buffer	BioLegend	422201
